# Supplementary material for: ALKBH5 Stabilized N6-Methyladenosine—Modified LOC4191 to Suppress E. coli-Induced Apoptosis
Source: Cells. 2023 Nov 10;12(22):2604. doi: 10.3390/cells12222604 (PMC10670315; doi:10.3390/cells12222604)
Supplement: Supplementary file 1 [file cells-12-02604-s001.zip › Table S1 Related primer sequences in RT-qPCR.pdf]

Table S1. Related primer sequences

gene	Primers Sequence (5′→3′)	
β-actin	F: TGCTGTCCCTGTATGCCTCT	R: GGTCTTTACGGATGTCAACG
IL-1β	F: TTCCATATTCCTCTTGGGGTAGA	R: AAATGAACCGAGAAGTGGTGTT
IL-6	F: CAGCAGGTCAGTGTTTGTGG	R: CTGGGTTCAATCAGGCGAT
TNF-α	F: CTTCTCAAGCCTCAAGTAACAAGC	R: CCATGAGGGCATTGGCATAC
METTL3	F: GGAACACTGCTTGGTTGGTG	R: GGTTGCACATTGTGTGGTCG
METTL14	F: TTGGAGCAAGGGTTCATCCG	R: CACTTTCAGCTCCCAACTGC
WTAP	F: CTCCGTCTGGAGAGGATTCA	R: CTGCGTGCAGATTCTTGCTG
ALKBH5	F: CCCATCCACATCTTCGAGCG	R: AGCAGCGTATCCACTGAGCAC
FTO	F: CTCCGTCTGGAGAGGATTCA	R: TGCTCCTTGGTTGCTAGTCG
