# Supplementary figures and images for: ALKBH5 Stabilized N6-Methyladenosine—Modified LOC4191 to Suppress E. coli-Induced Apoptosis
Source: Cells. 2023 Nov 10;12(22):2604. doi: 10.3390/cells12222604 (PMC10670315; doi:10.3390/cells12222604)

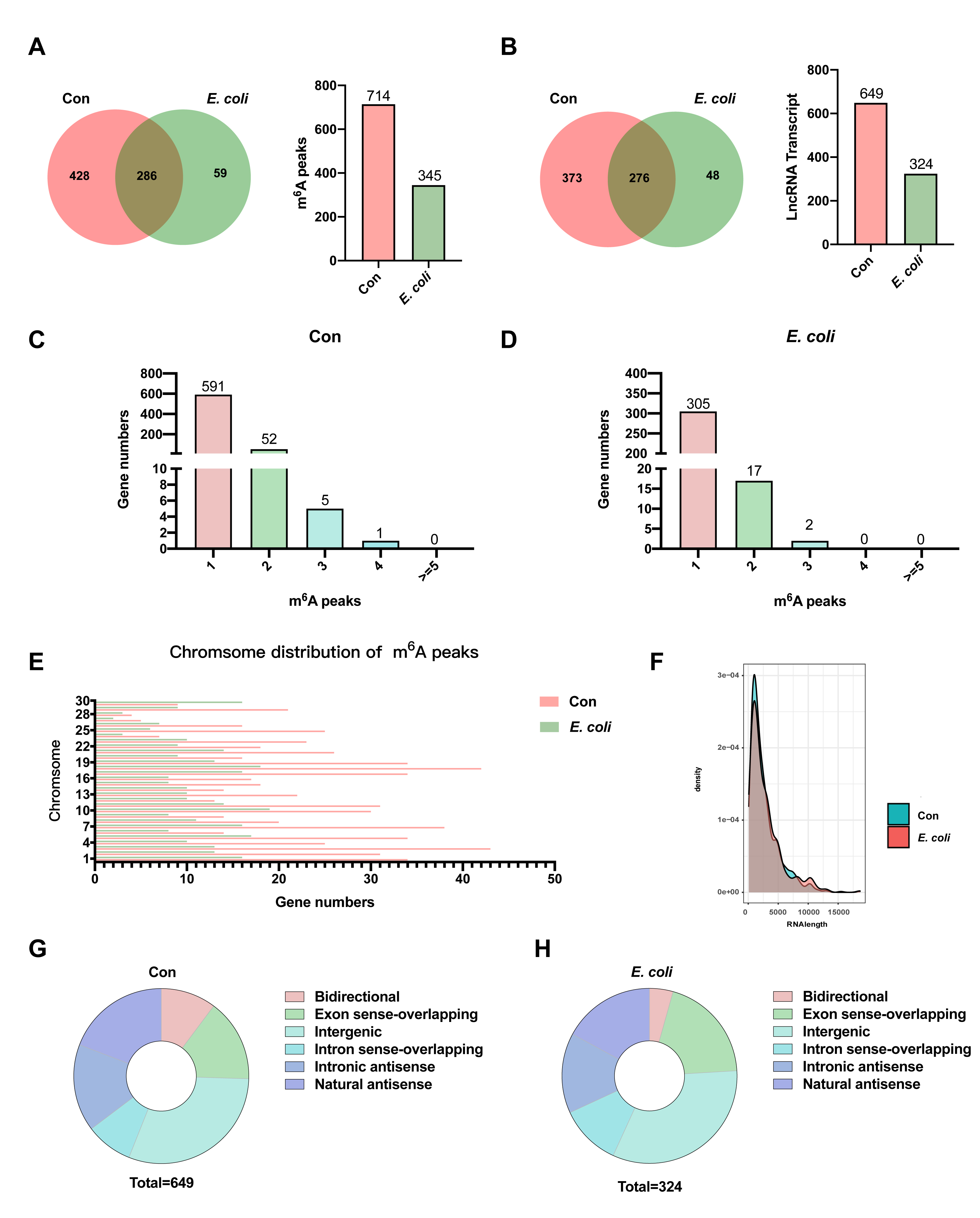

Supplement: Supplementary file 1 [file cells-12-02604-s001.zip › Figure S1.jpg]

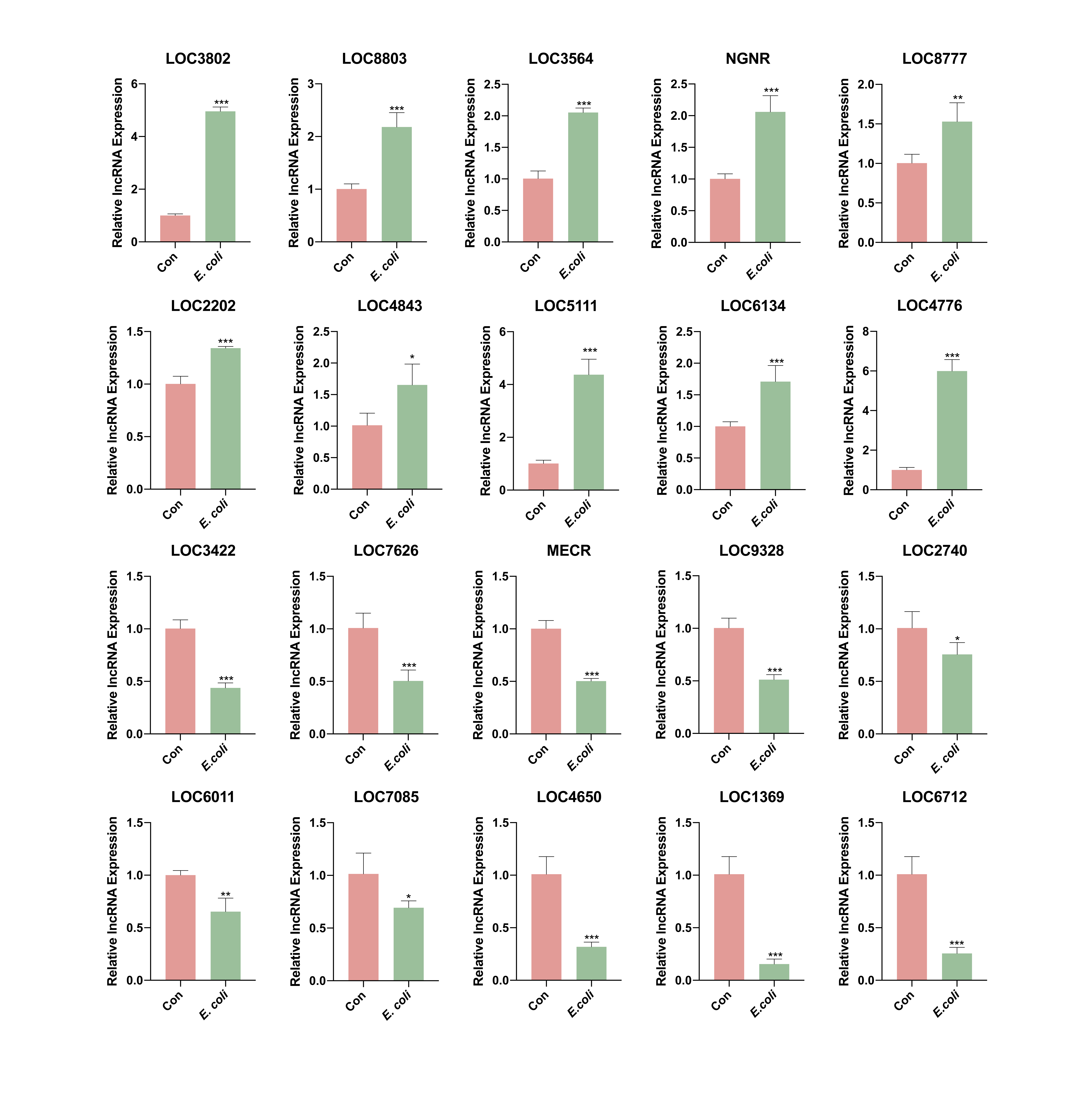

Supplement: Supplementary file 1 [file cells-12-02604-s001.zip › Figure S3.jpg]

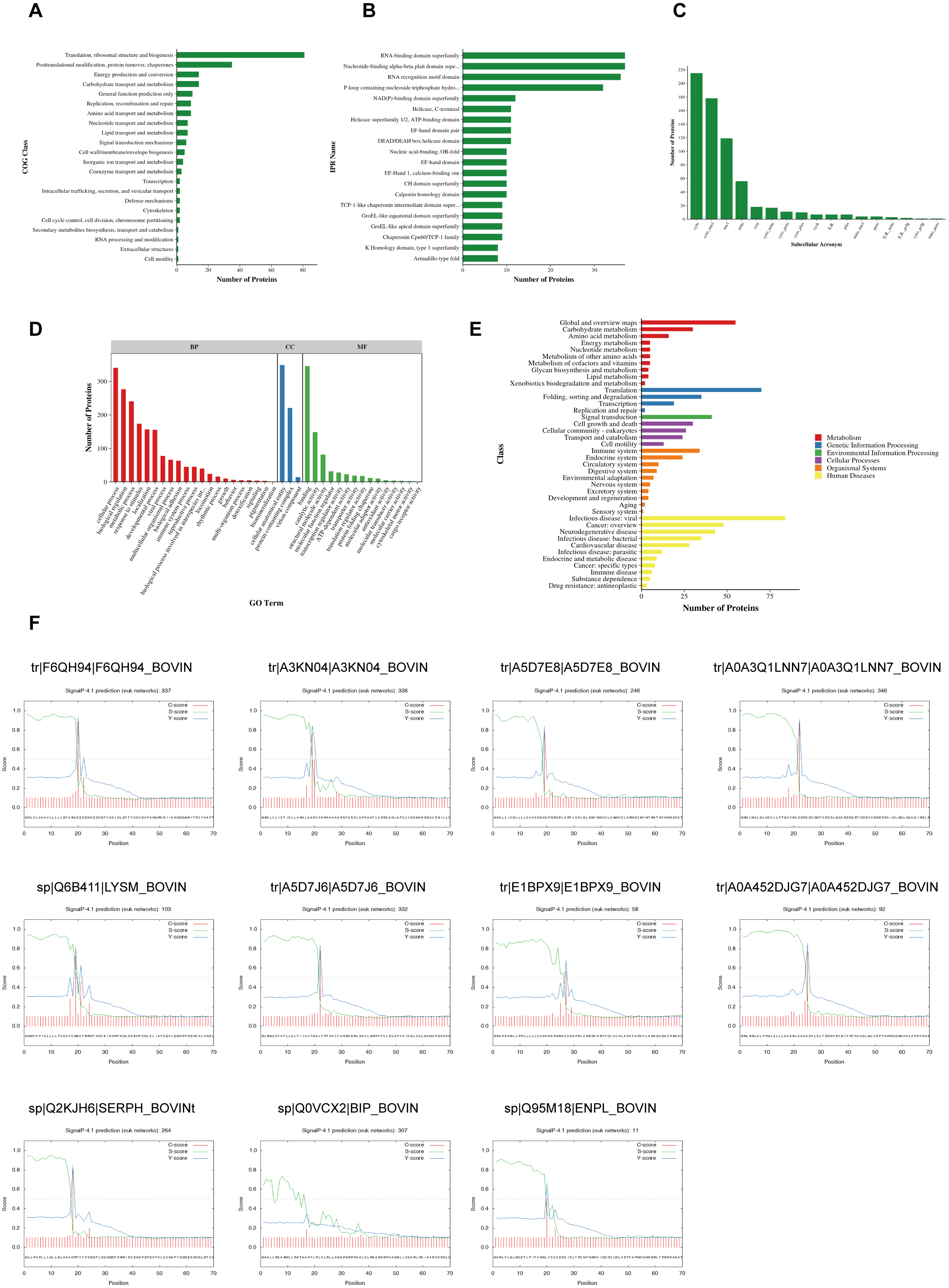

Supplement: Supplementary file 1 [file cells-12-02604-s001.zip › Figure S4.jpg]
